# Preparation and Performance Study of the Anodic Catalyst Layer via Doctor Blade Coating for PEM Water Electrolysis

**DOI:** 10.3390/membranes13010024

**Published:** 2022-12-24

**Authors:** Gaoyang Liu, Shanlong Peng, Faguo Hou, Xindong Wang, Baizeng Fang

**Affiliations:** 1 Department of Energy Storage Science and Technology, University of Science and Technology Beijing, 30 College Road, Beijing 100083, China; 2 Department of Metallurgical and Ecological Engineering, University of Science and Technology Beijing, 30 College Road, Beijing 100083, China

**Keywords:** PEM water electrolysis, membrane electrode assembly, catalyst layer, doctor blade coating, iridium oxide

## Abstract

The membrane electrode assembly (MEA) is the core component of proton exchange membrane (PEM) water electrolysis cell, which provides a place for water decomposition to generate hydrogen and oxygen. The microstructure, thickness, IrO_2_ loading as well as the uniformity and quality of the anodic catalyst layer (ACL) have great influence on the performance of PEM water electrolysis cell. Aiming at providing an effective and low-cost fabrication method for MEA, the purpose of this work is to optimize the catalyst ink formulation and achieve the ink properties required to form an adherent and continuous layer with doctor blade coating method. The ink formulation (e.g., isopropanol/H_2_O of solvents and solids content) were adjusted, and the doctor blade thickness was optimized. The porous structure and the thickness of the doctor blade coating ACL were further confirmed with the in-plane and the cross-sectional SEM analyses. Finally, the effect of the ink formulation and the doctor blade thickness of the ACL on the cell performance were characterized in a PEM electrolyzer under ambient pressure at 80 °C. Overall, the optimized doctor blade coating ACL showed comparable performance to that prepared with the spraying method. It is proved that the doctor blade coating is capable of high-uniformity coating.

## 1. Introduction

In order to reduce the consumption of fossil fuels and achieve carbon neutrality, hydrogen production based on the renewable energy sources is a sustainable alternative [1,2,3,4,5,6,7,8,9]. The proton exchange membrane (PEM) water electrolysis is considered to be one of the most promising technologies [10,11]. Compared with alkaline water electrolysis and the solid oxide water electrolysis, PEM water electrolysis has the advantages of small cell size, high efficiency and high current density [12,13]. Generally, the membrane electrode assembly (MEA) is the most important component that has a significant impact on the performance and lifetime of the PEM water electrolyzers. It is extremely desirable to develop the MEA with high-performance and long-life, good consistency and low-cost, for accelerating the commercialization of PEM [14,15].

During the process of the water electrolysis, the mass transport, electronic and proton transport related to the electrochemical reactions take place in the catalyst layer (CL) of the MEA [16,17]. The CL is a porous structured electrode consisting of catalyst particles and an ionomer, where stable solid-liquid reaction interfaces are established to provide effective electrocatalytic active sites [18]. The ionomer in the CL not only acts as a binder to maintain the microstructure, but also forms a contiguous network of the proton transport [19]. Many publications have reported that the improvement of electron and proton conductivity, as well as efficient gas-liquid transport inside the CL are of importance to promote the performance and stability of the MEA [20,21,22]. To date, a lot of investigations have been made on the catalysts with high catalytic activity and durability, but there is not much work has been done on the fabrication and optimization of the CLs.

Presently, the preparation of MEA for PEM water electrolysis mainly draws on the methods of PEM fuel cells [23]. The CL can either be coated onto the gas diffusion substrate or the PEM. Typically, the gas-diffusion electrode (GDE) is achieved by fabricating the CL directly on the gas diffusion layers (GDL), and the as-obtained anode GDE and the cathode GDE are then hot pressed on both sides of the PEM to form a 5-layer MEA [24,25]. Even though lots of advantages of the GDE based 5-layer MEA including good structural stability and high mass transport due to the formation of pores, and low thermal and electronic resistances, the GDE method is still restricted by several disadvantages [26]. First, the catalyst is easy to penetrate into the GDL during the preparation process, resulting in low catalyst utilization. In addition, the adhesion between CL and PEM is usually poor, which could result in high interfacial resistance. Alternately, the CL can be also coated onto the PEM to form a catalyst-coated membrane (CCM) based MEA [27]. Generally, the CCM method is the mainstream technology for preparing MEA, and features with reduced interfacial resistance between the CL and the PEM, and improved performance and catalyst utilization. Specifically, the CL are directly coated on the PEM or a substrate (e.g. polytetrafluoroethylene, polyimide, etc.) through a decal transfer process with various coating methods, such as laboratory techniques of ultrasonic spray coating [17,28], doctor blade coating [29], industrial-scale solution of slot-die [30], gravure [31], etc. It should be noted that the ultrasonic spray coating could achieve a reliable and reproducible CL with controlled loading, but is restricted by the high cost, time-consuming, and limited active area (5–500 cm^2^). Currently, there are few reports of industrial-scale solution focusing on the fabrication of CL for PEM water electrolysis. The most industrial-scale solutions such as coating methods (slot, gravure, doctor blade, etc.) are optimized to deposit CL on a substrate for PEM fuel cells.

However, the catalyst used in the anode of the PEM water electrolysis is iridium oxide (IrO_2_) based catalysts, and their particle size, surface area, morphology and hydrophilic as well as hydrophobic performance are completely different from the Pt based catalysts used in PEM fuel cell [18,32,33]. The lower surface area and larger particle size of IrO_2_ could result in differences in catalyst ink viscosity, and the interaction between the catalyst and ionomer. It is thus necessary to optimize the catalyst ink formulation and achieve the ink properties required to form an adherent and continuous layer through coating methods. In this study, IrO_2_ was used as an anode catalyst for the ink formulation and coating processing. The ink formulation (e.g., solvents and solids content) were adjusted to achieve the ink properties required to form CL with the doctor blade coating method, which is a widely used roll to roll (R2R) coating method capable of high-uniformity coating. The surface and cross-section morphologies and thickness, catalyst loading of the ACL, and their effects on the cell performance were fully examined.

## 2. Experimental

### 2.1. Materials

Nafion^®^ solution (5 wt%) and Nafion^®^ XL were supplied by Dupont (Shenzhen, China). Commercial 20 wt% Pt/C was received from Beijing Nonferrous Metal Research Institute (Beijing, China). All other chemical reagents were purchased from Sinopharm Chemical Reagent Beijing Co., Ltd. (Beijing, China). All chemicals were used as received without further purification in this work.

### 2.2. Doctor Blade Coating

For the anodic CL (ACL), the home-made IrO_2_ was prepared with the Adams method as the OER catalyst [16]. Firstly, a mixed solvent with different mass ratios of isopropanol and deionized water (IPA/H_2_O) was prepared, and then a homogeneous ink composed of IrO_2_, Nafion^®^ solution, and the mixed solvent were obtained by using a high-shear disperser (T25 Ultra Turrax, IKA, Guangzhou, China) for 15 min at 10,000 rpm in an ice ultrasonic bath. The ionomer to catalyst (I:C) mass ratios in the inks are shown in Table 1.

The ACL on the polytetrafluoroethylene (PTFE) substrate was prepared with a modified knife-over-edge coating method conducted on an automatic film coater with an adjustable doctor blade (MTIMSK-AFA-L, MTI Corporation, Suzhou, China). Different gaps between the doctor blade and the substrate (herein defined as the doctor blade thickness, 50–250 μm) as well as a width of 8 cm were used for coating. Prior to each doctor blade coating, 200 μL of ink was liner placed on the PTFE substrate and the doctor blade was mounted vertically. The doctor blade was then moved with a speed of 2 cm s^−1^ for coating. The CLs were dried in an air ventilation oven at 80 °C.

### 2.3. Spray Coating

For spray coating ACL [16] and cathode CL [34], a homogeneous ink composed of the IrO_2_, Nafion^®^ solution and isopropanol was sprayed on a PTFE substrate to form a thin ACL, and a homogeneous ink composed of 40 wt% Pt/C (Johnson Matthey, London, UK) as cathode catalyst, Nafion^®^ solution and isopropanol was sprayed on a PTFE substrate to form a thin cathode CL. The I/C mass ratios in the CLs were 6:1 for both the ACL and the cathode CL, and the loading of IrO_2_ was 1.3 mg cm^−2^ for the anode CL and 0.5 mg cm^−2^ 40 wt% Pt/C for the cathode CL.

### 2.4. Preparation of the CCM

The Nafion^®^ XL (Dupont) was successively pretreated in 5 wt% H_2_O_2_ solution (Analytically Pure, Beijing Chemical Factory, Beijing, China), distilled water, 0.5 mol L^−1^ H_2_SO_4_ solution and distilled water at 80 °C for 60 min of each step. The CCM was eventually obtained by transferring the CL from the PTFE substrate to the pretreated Nafion^®^ XL with a decal method. The hot-pressing process was conducted under the conditions of 135 °C, 75 kg cm^−2^ for 3 min. Various CCMs were prepared with the doctor blade coated ACLs and the sprayed cathode CLs (the Pt loadings were 0.2 mg cm^−2^). For comparison, CCMs were also prepared with the sprayed ACLs and the sprayed cathode CLs.

### 2.5. Characterizations

Zeiss SUPRATM55 field emission scanning electron microscopy (SEM, Berlin, Germany) was used to characterize the in-plane and the cross-sectional morphology of the sample at an accelerating voltage of 15 kV. The energy dispersive spectroscopy (EDS, Berlin, Germany) was used to analyze the elemental distribution, and the thicknesses of the CLs were analyzed by the change of the elemental content in the cross-sectional EDS line scan spectrum. Before analysis, the membrane was cracked in liquid nitrogen and then sputter coated with a fine Pt layer.

Metallographic microscope was used to observe the coating quality, which was conducted on the MX6R model metallographic microscope produced by Sunny Optics Technology Co., Ltd. (Beijing, China)

The IrO_2_ loadings of the anode CLs were measured using X-ray fluorescence spectroscopy (XRF) (Fischer-scope XDV-SDD, 50 kV, 50 W X-ray source, Seattle, DC, USA). Each anode CL was measured at 5 different locations to characterize the average loading.

Contact angle measurements were performed on a FTÅ 200 instruments (Beijing, China) and the contact angle of ink was further determined by fitting a mathematical expression to the shape of the drop and then calculating the slope of the tangent to the drop at the liquid-solid-vapor (LSV) interface line.

The performance test was performed in a home-made PEM electrolyzer under ambient pressure at 80 °C. MEA (with a 5.84 cm^2^ active area) was fabricated by placing the CCM between two carbon cloths. Distilled water was fed to the anode and cathode at a flow rate of 3 mL min^−1^. The cathode of the cell was used as both the counter and reference electrodes. Polarization curves were measured in constant current mode by increasing the current density from 0 to 2 A cm^−2^.

## 3. Results and Discussion

**Figure 1:**
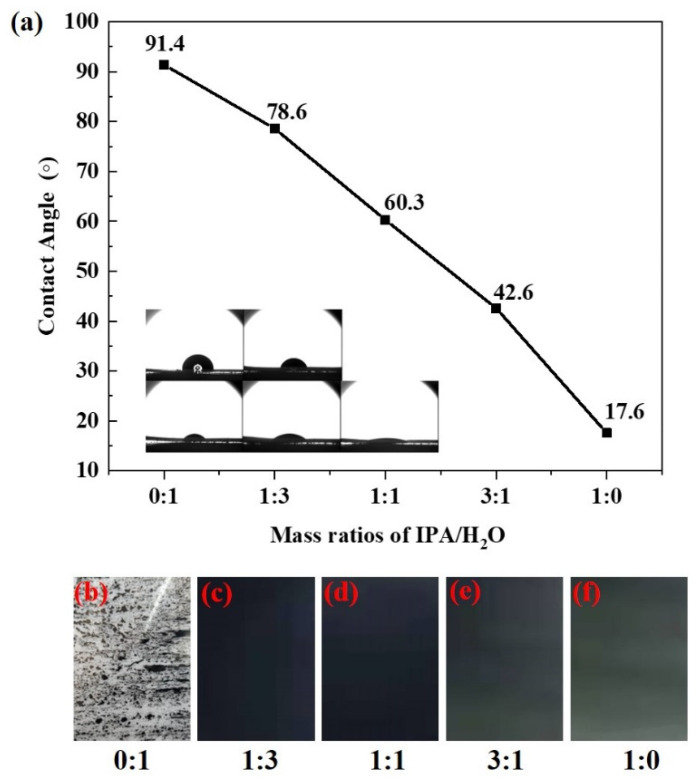
(**a**) the contact angle of inks with different IPA/H_2_O ratios on the PTFE membrane, (**b**–**f**) photographs of doctor blade coating ACL prepared with different IPA/H_2_O, and the solid content was 20 wt%, the doctor blade thickness was 150 μm.

In order to coat the ink on the PTFE membrane effectively and uniformly, the contact angle of the ink on the PTFE membrane is particularly important, which largely determines the quality of the coating layers [35,36]. Figure 1a shows the contact angle of inks with different mass ratios of IPA/H_2_O on the PTFE membrane. It can be seen that the contact angle decreases with the increase in IPA content, and the contact angles with pure water and pure IPA are 91.4° and 17.6°, respectively. The contact angle decreases from 78.6° for a 1:3 mixed ink to 60.3° for a 1:1 mixed ink and 42.6° for a 3:1 mixed ink. It should be noted that the hydrophilicity or hydrophobicity of the ink is closely related with the wettability and the surface tension of the wet coating on the PTFE, which will affect the loading and the quality of the coatings [37]. If an ink with low IPA content is used, the wettability of the ink on the PTFE film is poor due to the surface tension. The ink will appear as shrinkage during the volatile drying process, and as a result the obtained coating appears with extremely uneven size streaks. Figure 1b shows the samples coated with pure water, and only small, condensed streaks of catalyst were observed after drying. The good wettability of the ink on the PTFE film should also be avoided. Figure 1f shows the samples coated with pure IPA. It can be seen that the samples coated with pure IPA show an asymmetrical coating layer from a dark color to a light color. Figure 1d,e show the coating layers prepared with different IPA/H_2_O ratios (1:3, 1:1, 3:1). Improved coating quality can be obtained, and there were no visible pinholes or bubbles as well as large voids presented in the coating. Therefore, the range of IPA/H_2_O ratios could be possible for the doctor blade coating.

**Figure 2:**
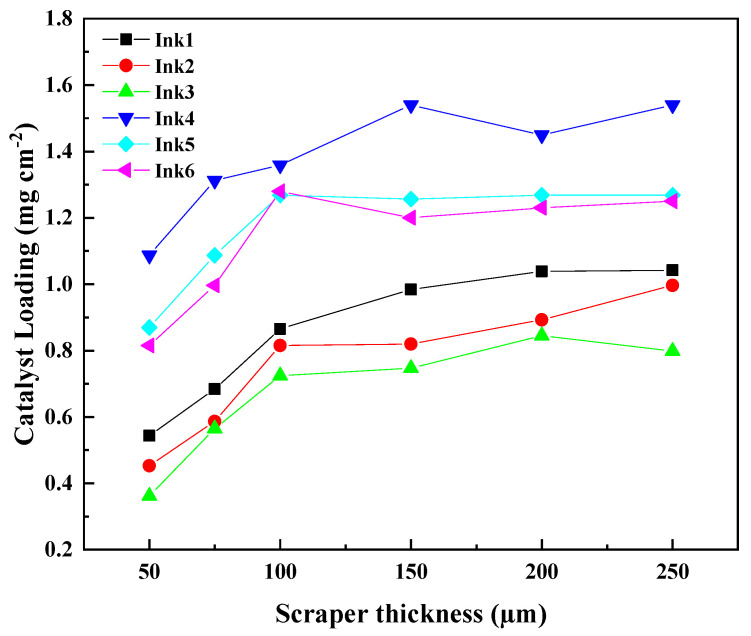
The IrO_2_ loading for the doctor blade coating ACL prepared with different ink as a function of the doctor blade thickness.

Figure 2 shows the IrO_2_ loading of the ACLs prepared with the doctor blade coating with different inks and different doctor blade thicknesses. It can be seen that the IrO_2_ loading increases significantly with the increase in doctor blade thickness when it is below 150 µm for all inks. When the doctor blade thickness is greater than 150 μm, only a slight increase in the IrO_2_ loading before reaching a plateau can be observed for both inks with 10 wt% solids and 20 wt% solids. The ability of doctor blade thickness to tune the loading could be limited by factors such as the height and the surface tension of the wet coating [36,37]. As for the effect of solid content of the ink, it can be clearly seen that the ACL prepared with 20 wt% solid content has a higher IrO_2_ loading than that with 10 wt% solid content, indicating that the usage of higher solid content could promote the higher IrO_2_ loading. While higher solid content of 30 wt% could result in ropiness ink with low velocity, and finally poor coating. The minimum IrO_2_ loading is 0.36 mg cm^−2^ and the highest is 1.04 mg cm^−2^ when the solid content is 10 wt%, and the lowest load is 0.82 mg cm^−2^ and the highest is 1.54 mg cm^−2^ when the solid content is 20 wt%. Interestingly, the IrO_2_ loading can be also affected by the IPA/H_2_O ratios, it can be found that the IrO_2_ loading is correlated with the water content or the hydrophilicity of the inks. For both 10 wt% solids and 20 wt% solids, the higher IPA/H_2_O ratios tend to lead to lower IrO_2_ loading. Because of the stronger hydrophilicity, the ink with higher IPA/H_2_O ratio is more easily spreading on the PTFE membrane, and less catalyst will be deposited per unit area. Overall, it can be concluded that the IrO_2_ loading can be controllable via adjusting the solids content, and the hydrophilicity of the inks as well as the doctor blade thickness.

**Figure 3:**
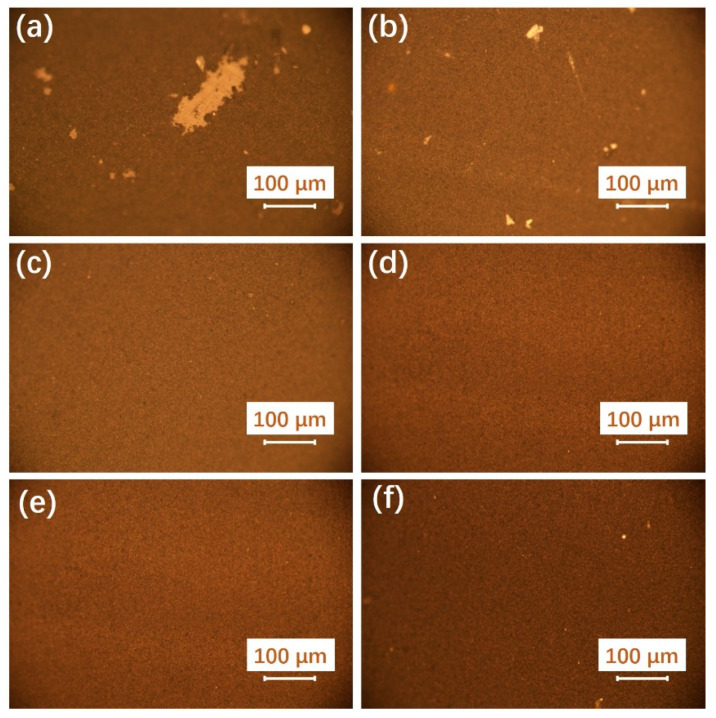
The metallographic images of the doctor blade coating ACLs with different doctor blade thicknesses: (**a**) 50 µm, (**b**) 75 µm, (**c**) 100 µm, (**d**) 150 µm, (**e**) 200 µm, (**f**) 250 µm, and all the samples were prepared with the inks of same IPA/H_2_O ratio (1:3) and the solid content (20 wt%).

In addition to the tuning of the IrO_2_ loading, the coatability of each ink as well as the uniformity and quality of the coating with different doctor blade thicknesses and inks of different IPA/H_2_O ratios were further investigated in this work. Figure 3 shows the metallographic images of the samples coated with different doctor blade thicknesses, and all the samples were prepared with the same ink with the IPA/H_2_O ratio (1:3) and the solid content (20 wt%). It can be seen that the uniformity and the quality could be significantly affected by the doctor blade thickness. There exist some large voids and pinholes in the coating when the thickness of doctor blade is below 100 µm (Figure 3a,b). It could be related to the catalyst and ionomer aggregated particles with a larger size than the doctor blade thickness, leading to the friction effect during the doctor blade coating process. Compared with the coating obtained with the doctor blade thickness of 50 and 75 µm, the coatings obtained with the doctor blade thickness from 100 to 200 µm are visually uniform and no voids are observed (Figure 3c–e). It indicated that there should be a good match between the doctor blade thickness and the surface tension, which results in a uniform wet coating on the PTFE. While for the coating with the doctor blade thickness of 250 µm (Figure 3f), there exist some tiny pinholes on the surface of the coating which were caused by the small bubbles or streaks of catalyst during the drying process. Therefore, based on the previous results, it can be concluded that the doctor blade thickness had a large influence on both the IrO_2_ loading and the quality of the coating when the doctor blade thickness is low, but the influence on both the IrO_2_ loading and the quality of the coating is tiny when the doctor blade thickness is high.

**Figure 4:**
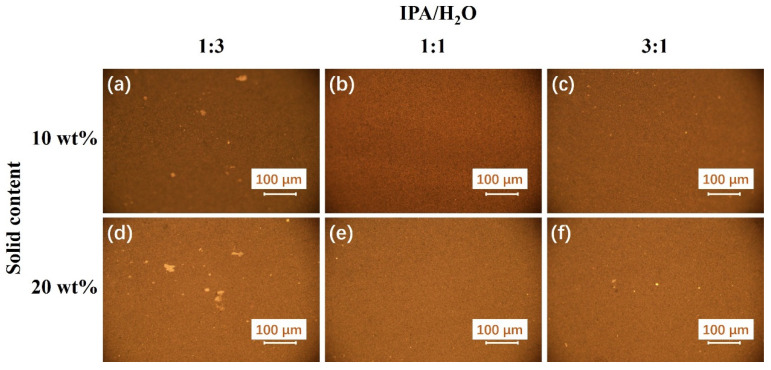
The metallographic images of the doctor blade coating ACLs with different IPA/H_2_O ratios and the solid contents, and the doctor blade thickness is 150 µm for all the samples.

The uniformity and quality of the coating can also be affected by the IPA/H_2_O ratio and the solid content of the ink [38,39]. Figure 4 presents the metallographic images of the samples as a function of the IPA/H_2_O ratios and the solid contents of the ink. All the doctor blade thickness was 150 µm. Most of the coatings look uniform and opaque. Obviously, the coatings with a solid content of 20 wt% are visually darker than the coatings with a solid content of 10 wt%, which reflected the higher IrO_2_ loading which is not good for coating. As for the influence of the IPA/H_2_O ratios, the coating with IPA/H_2_O (1:3) presents the most voids or pinholes regardless of the solid content. Typically, there are the most irregularities for the coating with IPA/H_2_O (1:3) and the solid content (10 wt%). The poor uniformity and quality of the coating with IPA/H_2_O (1:3) could be ascribed to the poorer dispersion of the catalyst and ionomer aggregated particles in the heterogeneous ink. For the coating with IPA/H_2_O (1:1), it was observed the best uniformity and quality of the coating without any voids, pinholes, or cracks. When the IPA/H_2_O ratio is 1:3, there are some tiny irregularities, such as pinholes and uneven granularities. As shown by the previous results, the good wettability and the strong volatility of the ink with high isopropanol content could result in the small bubbles or streaks of catalyst during the dewetting process. Overall, the uniformity and quality of the coating is significantly affected by the IPA/H_2_O ratio of the ink, as well as the doctor blade coating parameter such as the doctor blade thickness. The solid contents have minimal influence on the uniformity and quality of the coating when the solid content is below 20%, but the adjustment of the solid contents is an effective way for tuning the IrO_2_ loading. Generally, based on the above experimental results, both the tunable IrO_2_ loading range from 0.36 to 1.54 mg cm^−2^, and high uniformity and quality of the coating can be achieved. The doctor blade coating is expected to be an effective and low-cost method for the ACL preparation compared with the current commonly used spraying coating. MEAs with a larger active area could be produced in a very short term via the optimized doctor blade coating.

**Figure 5:**
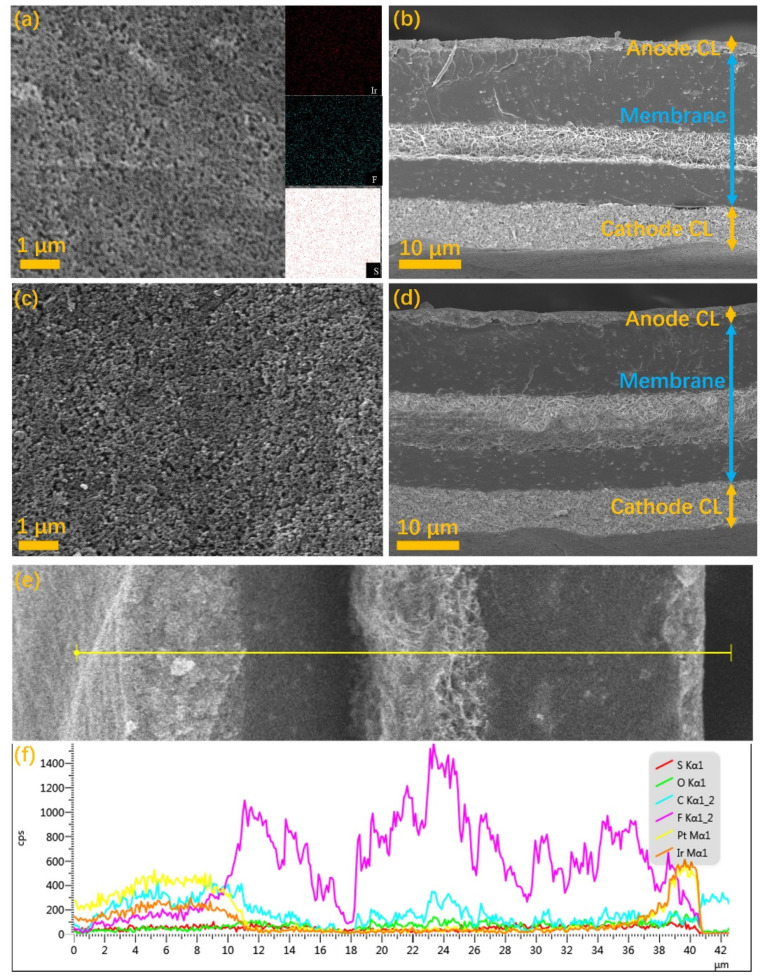
The in-plane SEM images and the cross-sectional SEM images of the samples prepared with doctor blade coating (**a**,**b**) and spraying coating (**c**,**d**), the magnified cross-sectional SEM images (**e**), the yellow line is the EDS line scan direction and the corresponding EDS line scan spectra (**f**).

Figure 5a,b and Figure 5c,d present the in-plane SEM images of ACL coatings and the cross-sectional SEM images of the MEAs prepared with the doctor blade coating and spraying coating, respectively. From the surface morphology of the ACLs, uniform porous structure without obvious protrusion or groove could be observed for the ACLs by the two ACL methodologies, and the size of the catalyst and ionomer aggregated particles did not differ obviously. However, it can be seen that the ACL prepared with the spraying coating features with more porosity as well as uniform and flat surface than that with doctor blade coating. The pore size distribution is not quite uniform and more dense areas can also be observed for the ACL with doctor blade coating. The inserts present the EDS elemental mapping of Ir, S and F. It can be seen that the distribution of Ir, S and F was relatively uniform, and the distribution of Ir and F element has a high coincidence. It indicated that the homogeneity of the ink for doctor blade coating was fully demonstrated. From the cross-sectional SEM images of the MEAs with different ACLs, it can be seen that the thickness of ACL with the doctor blade coating is almost the same with the ACL with spraying coating, and more uniform pore size distribution was further observed for the ACL by spraying coating. It should be pointed out that there were no noticeable cracks or strips between the ACLs and the MEAs for both the ACLs after the hot-pressing, indicating that the ACL by doctor blade coating could be used for the decal fabrication of the MEA. Figure 5e,f present the magnified cross-sectional SEM images of the MEA with the doctor blade coated ACL, and the EDS line scan spectra of elemental distribution, respectively. Typically, the content of Ir, Pt and C, S and F are indicators of the ACL (IrO_2_ catalysts), cathode CL (Pt/C catalysts), and the PEM ((C_7_HF_13_O_5_S C_2_F_4_)_x_), respectively [40,41]. The thickness of different layers can be confirmed by the change of the content of the elemental indicators. The membrane used in this work was from Nafion^®^ XL, which was a reinforced membrane with a thickness of 27.5 μm. The thickness of the ACL with the doctor blade coating was further confirmed to be around 2 µm. It should be noted that the surface morphology, the porous structure as well as the thickness of the ACL are of significant importance to the diffusion of reactants and products as well as the electronic and protonic transport, which result in different performance and stability when applied in PEM water electrolyzers.

**Figure 6:**
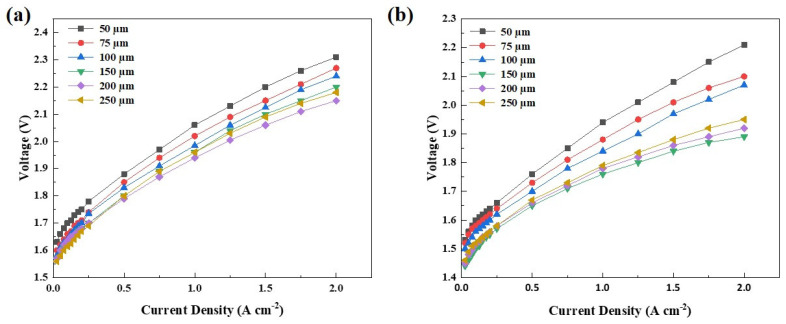
The polarization curves for the MEAs with doctor blade coating ACLs of different doctor blade thicknesses: (**a**) ink1, (**b**) ink4.

Figure 6 shows the polarization curves under ambient pressure and 80°C with the ACLs prepared with the doctor blade coating corresponding to ink1 (10 wt% IrO_2_, IPA/H_2_O = 1:1) and ink4 (20 wt% IrO_2_, IPA/H_2_O = 1:1), respectively. It is clear from Figure 6a-b that cell performance is improved with the increase in the doctor blade thickness until 200 µm for ink1 and 150 µm for ink4. According to the previous results, the better cell performance can be ascribed to the increasing IrO_2_ loading with the increase in doctor blade thickness. When the doctor blade thickness was higher than 200 µm for ink1 and 150 µm for ink4, only slight increase in the IrO_2_ loading can be observed. It has been reported that the higher catalyst loading could be not conducive to the mass transport of the liquid and the generated oxygen, and also result in a higher ohmic resistance due to a larger thickness of the CL. Typically, the best cell performance was obtained with ink1-200 µm and ink4-150 µm, respectively. The IrO_2_ loading of ACL largely determines the cell performance and could be tunable via the parameters of the ink and coating process.

**Figure 7:**
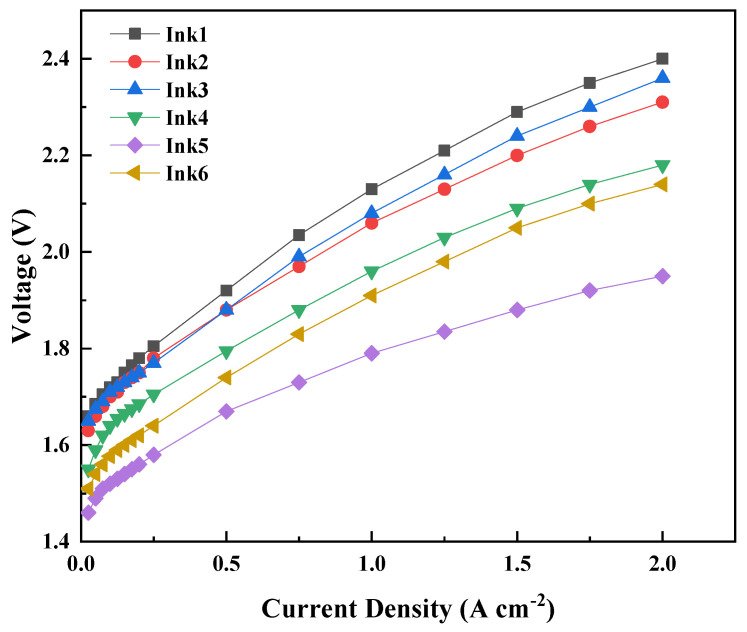
The polarization curves for the MEAs with doctor blade coating ACLs of different inks.

Figure 7 shows the polarization curves of the ACL prepared by using different inks and the doctor blade thickness was 150 µm. It can be clearly seen that the cell performance with inks 4–6 is much better than that with inks 1–3 mainly due to the higher IrO_2_ loading when using inks with higher solid content. Because the uniformity and quality of the coating could be affected by the IPA/H_2_O ratios, the cell performance of different inks was verified to be significantly influenced. The best cell performance was obtained with IPA/H_2_O (1:1) of the ink. When the current density was 2 A cm^−2^, the performance of membrane electrodes prepared with different inks was 2.36, 2.31, 2.0, 2.18, 2.14 and 1.95 V, respectively. It was found that the ACL prepared with the Ink5 (20 wt% IrO_2_, IPA/H_2_O = 1:1 alongside a doctor blade thickness of 150 µm had the best performance.

**Figure 8:**
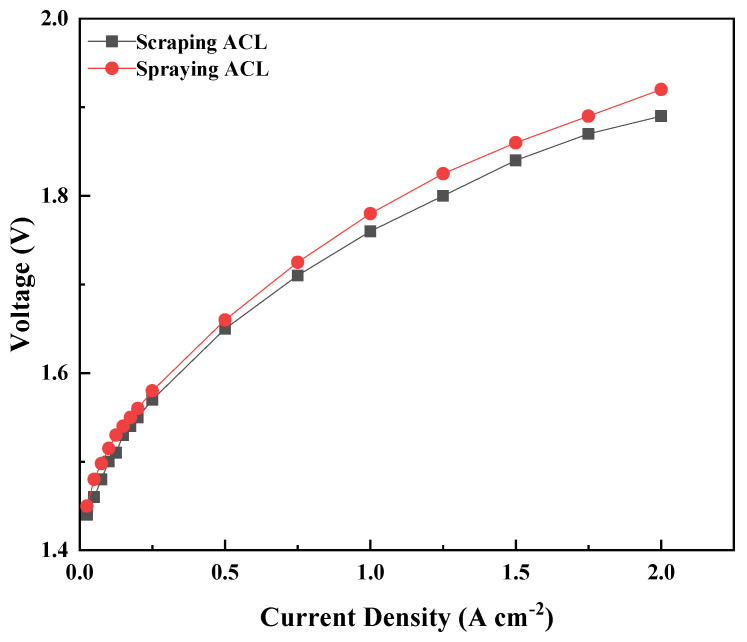
The polarization curves for the MEAs with doctor blade coating ACL and spraying ACL. The IrO_2_ loading of ACL was 1.3 mg cm^−2^.

Figure 8 shows the polarization curves of the MEAs with the ACL with the doctor blade coating and spraying coating. For the ACL prepared with the doctor blade coating method, the IrO_2_ loading was 1.3 mg cm^−2^. In order to maximize the influence of other factors, the same IrO_2_ loading of ACL prepared with spraying was also achieved by controlling the spraying time (the amount of ultrasonic ink). In addition, the hot-pressing time, hot pressing temperature and pressure were the same, and the mass ratio of IrO_2_ and Nafion in the ink was also consistent. From the polarization curves, it can be clearly seen that the ACL prepared with the doctor blade coating method has comparable performance to the ACL prepared with the spraying method. When the current density is 1 A cm^−2^, its voltage is 1.76 V and 1.78 V, respectively. It is further proved that the spraying method could be an effective and low-cost technology for MEA fabrication.

## 4. Conclusions

In this study, the ACLs for PEM water electrolysis were successfully prepared with the doctor blade coating method, and the MEA was then formed with sandwich-type stacking structure via hot-pressing decal method. The effects of both the composition of the ink (solid content, the IPA/H_2_O ratio) and the doctor blade thickness on the IrO_2_ loading as well as the uniformity and quality of the doctor blade coating ACL were explored and optimized. It was found that the hydrophilicity of the ink was directly affected by the IPA/H_2_O ratio in the ink, which resulted in different quality of the doctor blade coating ACLs. While the IrO_2_ loading (0.36 to 1.54 mg cm^−2^) and the quality of the doctor blade coating ACL can be controllable via adjusting the solids content, and the IPA/H_2_O of the inks as well as the doctor blade thickness. Both the in-plane surface and the cross-sectional morphology of the doctor blade coating samples and the spraying samples were further characterized with SEM, and the porous structure as well as an optimized thickness (around 2 µm) of the doctor blade coating ACL were confirmed. The MEAs with different doctor blade coating ACLs were examined in a PEM electrolyzer under ambient pressure at 80 °C. The IrO_2_ loading of the ACL as well as the quality of the doctor blade coating ACL significantly affected the cell performance, and the ACL prepared with ink5 (20 wt% IrO_2_, IPA/H_2_O = 1:1, a doctor blade thickness of 150 µm) showed the best performance. Interestingly, the optimized doctor blade coating ACL showed comparable performance to that prepared with the spraying method, indicating that the doctor blade coating method could be an effective and low-cost technology for MEA fabrication.

## Figures and Tables

**Table 1:** Composition of the different inks.

Ink No.	Solid Content (IrO_2_ wt%)	IPA/H_2_O	I/C
Ink 1	10	1:3	6:1
Ink 2	10	1:1	6:1
Ink 3	10	3:1	6:1
Ink 4	20	1:3	6:1
Ink 5	20	1:1	6:1
Ink 6	20	3:1	6:1

## Data Availability

Data will be available upon request from the corresponding authors.
